# Dietary Monounsaturated Fatty Acids Intake and Risk of Skin Photoaging

**DOI:** 10.1371/journal.pone.0044490

**Published:** 2012-09-06

**Authors:** Julie Latreille, Emmanuelle Kesse-Guyot, Denis Malvy, Valentina Andreeva, Pilar Galan, Erwin Tschachler, Serge Hercberg, Christiane Guinot, Khaled Ezzedine

**Affiliations:** 1 CE.R.I.E.S. (Research Centre on Human Skin Founded by CHANEL), Neuilly sur Seine, France; 2 UMR U557 INSERM/U1125 INRA/CNAM, University Paris 13/Centre of Research on Human Nutrition Ile de France, Paris/Bobigny, France; 3 Department of Internal Medicine and Tropical Diseases, Hôpital Saint-André, Bordeaux, France; 4 Department of Dermatology, Medical University of Vienna, Vienna, Austria; 5 Department of Public Health, Hôpital Avicenne, Bobigny, France; 6 Computer Science Laboratory, University François Rabelais, Tours, France; 7 Department of Dermatology, Hôpital Saint-André, Bordeaux, France; The University of Queensland, Australia

## Abstract

**Background:**

Intake of monounsaturated fatty acids has been reported to reduce oxidative stress, insulin resistance and related inflammatory processes and may thus protect from skin photoaging. The objective of this study was to investigate the association between the risk of photoaging, monounsaturated fatty acids intake and the sources of monounsaturated fatty acids.

**Methodology/Principal Findings:**

A cross sectional study was conducted within the framework of the SUVIMAX cohort. The survey included 1264 women and 1655 men aged between 45 and 60 years old. Dietary monounsaturated fatty acids intakes were estimated by dietary source through at least ten 24-h diet records completed during the first 2.5 years of the follow-up period. Severity of facial skin photoaging was graded by trained investigators at baseline during a clinical examination using a 6-grade scale illustrated by photographs. A lower risk of severe photoaging was associated with higher intakes of monounsaturated fatty acids from olive oil in both sexes. Strikingly, no association was found with intake of monounsaturated fatty acids from animal sources whether from dairy products, meat or processed meat.

**Conclusion/Significance:**

These findings support the beneficial effect of dietary olive oil or healthy diet habits associated with olive oil consumption on the severity of facial photoaging.

## Introduction

In the past century, life-expectancy has increased in most developed countries [1]. Changes to the appearance of the skin represent a visible sign of tissue alteration that occurs with age [2]. More specifically, skin aging is an important public health issue as it may result in the development of a large range of morbidities including non melanoma skin cancers [3].

Skin aging is driven by both intrinsic and extrinsic factors. Intrinsic aging, also referred to as chronological skin aging, is an ineluctable process [4], due to genetically determined loss of cell function with age. Intrinsic skin aging is characterized by fine wrinkles, and dry, thin and pallid skin [5], [6]. Extrinsic skin aging overlays intrinsic aging and is dependent on environmental and behavioral factors, in particular sun exposure. Extrinsic aging is characterized by solar elastosis, actinic keratosis, pigmentation and vascular abnormalities. The ultimate stage of this process is skin cancer, namely basal cell carcinomas and squamous cell carcinomas [7]–[9]. The main factor responsible for extrinsic aging is ultraviolet radiation and is thus referred to as skin photoaging. Skin damage, which may in part be reversible, is mainly driven by the production of reactive oxygen species (ROS) and related inflammation occurring in response to cumulated or intermittent intense sun exposure. Exposure to UVB damages DNA directly through generation of cyclobutane pyrimidine dimers and 6-4 photoproducts in keratinocytes and melanocytes, whereas UVA damages more indirectly through generation of ROS, leading to lipid peroxidation, activation of transcription factors (NF-kB and AP-1) and DNA strand breaks.

Numerous studies have focused on the possible role of diet in the capacity of the skin to resist damage induced by UV radiation [10]. Although the skin is a major fat storage organ in humans, data on the impact of lipid intake on skin physiology are limited. Low fat intake has been proposed to protect from photodamage [11]. In particular, monounsaturated fatty acids (MUFA) have been reported to reduce oxidative stress, insulin resistance and related inflammation [12]–[15].

In this context, we have performed an analysis within the framework of the SU.VI.MAX cohort designed to explore possible associations between the severity of facial skin photoaging, MUFA intake and the sources of MUFA. Photoaging was measured using a 6-grade scale specially developed and validated for assessing the overall severity of photodamage, including pigmentation abnormalities, wrinkling and tissue slackening [16].

**Figure 1:**
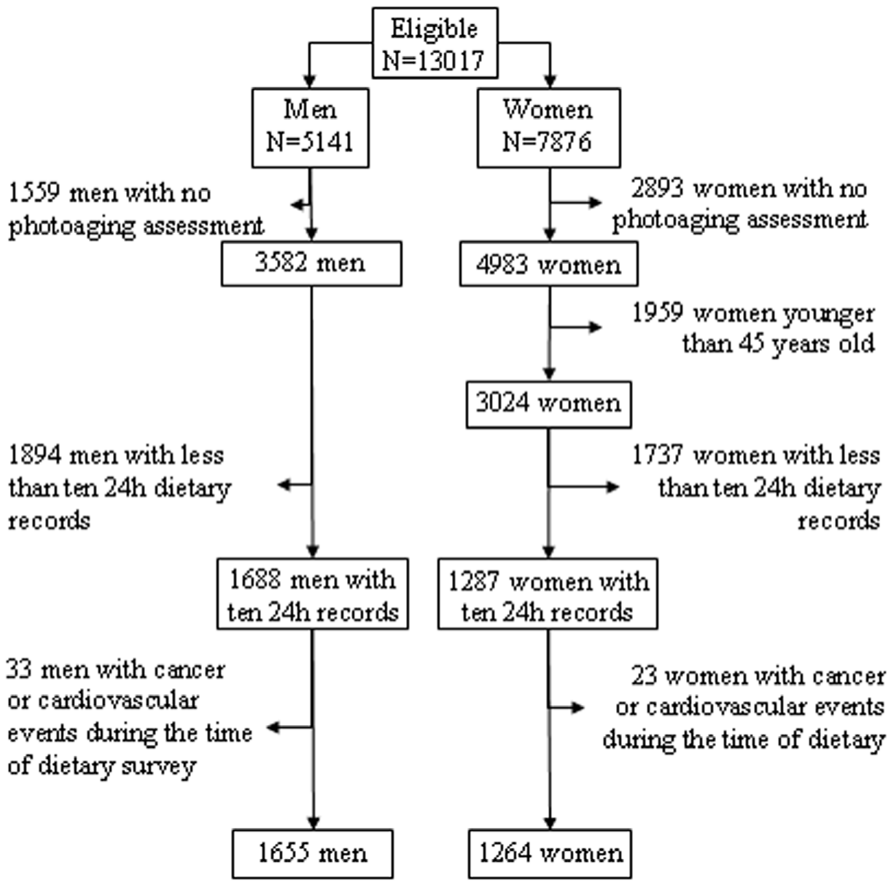
Flow chart of participants from the SU.VI.MAX study cohort retained in the analysis.

## Materials and Methods

### Study Population

Subjects were participants in the SU.VI.MAX (*Supplémentation en Vitamines et Minéraux Antioxydants*) study, a double blind, placebo-controlled primary prevention trial evaluating the effect of antioxidant supplementation (a mixture of vitamin C, vitamin E, β-carotene, zinc, and selenium) on the incidence of ischemic heart diseases and of cancers in a population of adult men and women. A total of 13,017 volunteers, 7876 women and 5141 men were included in 1994–1995 with a planned follow-up of eight years. Men were aged 45–60 years and women 35–60 years at enrolment. The design, objectives and methodology of the study have been described *in extenso* elsewhere [17]. All subjects gave their informed written consent to the study. The study was approved by *ad hoc* ethical committees, the “*Comité Consultatif de Protection des Personnes dans la Recherche Biomédicale*” (CCPPRB no. 706, Cochin Hospital, Paris, France), and the “*Commission Nationale de l’Informatique et des Libertés*” (CNIL no. 334641). The study was registered at clinicaltrials.gov as NCT00272428 [18].

**Table 1:** Demographic, medical and behavioral characteristics according to photoaging severity.

		Women (n = 1264)					Men (n = 1655)				
		Photoaging grades					Photoaging grades				
		1–2	3	4	5–6		1–2	3	4	5–6	
Characteristics		(n = 193)	(n = 555)	(n = 402)	(n = 114)	P-value	(n = 212)	(n = 744)	(n = 574)	(n = 125)	P-value
Age (years)		48.8 (3.5)	50.6 (4.1)	53.2 (4.0)	56.5 (3.7)	<0.0001	48.3 (4.2)	51.2 (4.4)	53.9 (3.9)	57.9 (2.6)	<0.0001
BMI (kg/m^2^) (%)	<25	76	79	72	72	0.13	54	50	50	53	0.60
	[25–30[	16	15	22	20		40	44	44	38	
	> = 30	8	5	6	8		6	6	6	8	
Phototype* (%)	I-II	3	3	6	5	0.18	2	2	1	5	0.0001
	IIIa	14	12	10	9		12	7	8	6	
	IIIb	52	53	49	52		37	45	40	35	
	IV	29	25	29	27		45	41	42	42	
	V–VI	1	5	5	6		4	4	9	13	
Lifetime sun	None – Low	8	9	10	11	0.88	13	10	13	8	0.26
exposure* (%)	Moderate	58	58	59	55		59	60	61	58	
	High	33	28	29	30		28	29	26	34	
Overall physical	Irregular	29	24	25	16	0.10	22	24	21	14	0.21
activity* (%)	<1 h/day	34	34	36	31		27	24	23	19	
	≥1 h/day	37	42	40	54		52	53	56	66	
Smoking habits* (%)	Never smokers	62	66	63	75	0.24	34	38	35	35	0.67
	Former smokers	26	25	27	17		55	52	55	50	
	Smokers	11	8	9	8		11	11	10	15	
Hormonal status and	Non-menop	74	59	37	13	<0.0001					
MHT intake* ^,^ ‡ (%)	Menop with MHT	20	30	46	56						
	Menop without MHT	6	12	16	31						
Educational	Elementary school	22	23	23	25	0.38	27	25	25	31	0.0181
level* (%)	Secondary school	45	41	48	47		36	33	36	47	
	University or equivalent	33	35	29	27		37	41	39	22	
Geographic	North of France	67	66	72	75	0.12	71	67	65	77	0.0267
location† (%)	South of France	33	34	28	25		29	33	35	23	

Values are expressed as means (SD) or percentages. Differences in demographic, medical and behavioral characteristics between photoaging grades were examined using analysis of variance for continuous variables (age) and the chi^2^ test for categorical variables.

* Due to possible missing values the sum of the cell frequencies can be smaller than the total indicated in the top of the columns.

† France has been arbitrarily divided in north and south using the northern frontier of Aquitaine, Limousin, Auvergne, and Rhône-Alpes regions.

‡ MHT: menopausal hormone therapy.

### Dietary Assessment

In order to take into account possible seasonal and weekly intra-individual variations in dietary intake, subjects were asked to complete a 24-h dietary record every two months from the inclusion to the end of the study for a total of six records per year (two weekend days and four weekdays per year). Dietary data were collected using the Minitel Telematic Network, a small terminal that was widely used as an adjunct to the telephone in France at the beginning of the study in the 1990s. This 24h record included about 900 items relating to food and drink for each of three meals (breakfast, lunch, and dinner) and for four other possible occasions for food intake (snacks). For each item, the subjects were requested to indicate the portion size consumed. To improve the quality of data collected, subjects received an instruction manual at the start of the study, including photographs of three portion sizes of about 250 foods and drinks. The use of the manual has been validated elsewhere [19]. The questionnaire also included questions on the type of oil or fat used for seasoning and cooking. Other details of these diet diaries have been published previously [17], [20]. Subjects who completed at least ten records over a period of 2.5 years after inclusion were selected for this analysis. Ten records were considered to be sufficient to estimate the individual intake of monounsaturated fatty acids (FA) with acceptable accuracy [21]. Finally, all subjects who developed a cancer or a cardiovascular event during the course of the dietary survey (2.5 years) were not included in the analysis (Fig. 1).

**Table 2:** Dietary factors according to photoaging severity.

	Women (n = 1264)					Men (n = 1655)				
	Photoaging grades					Photoaging grades				
	1–2	3	4	5–6		1–2	3	4	5–6	
Dietary factors	(n = 193)	(n = 555)	(n = 402)	(n = 114)	P-value	(n = 212)	(n = 744)	(n = 574)	(n = 125)	P-value
Monounsaturated fat (% TEI)	15.5 (2.4)	15.5 (2.8)	14.8 (3)	15.1 (3.4)	0.0106	14.8 (2.9)	14.7 (2.9)	14.5 (2.8)	13.9 (2.8)	0.0005
Monounsaturated fat from										
Dairy (%TEI)	4.2 (1.7)	4.2 (1.7)	4.3 (1.7)	4.4 (1.5)	0.73	4.1 (1.5)	4.0 (1.6)	4.0 (1.8)	4.0 (1.5)	0.61
Meat (%TEI)	1.1 (0.8)	1.0 (0.9)	1.0 (0.8)	1.0 (0.9)	0.12	1.3 (1.1)	1.2 (0.9)	1.1 (0.9)	1.1 (0.8)	0.28
Processed meats (%TEI)	1.1 (1.1)	1.1 (1.3)	1.0 (1.3)	1.1 (1.4)	0.35	1.6 (1.5)	1.4 (1.5)	1.4 (1.4)	1.4 (1.6)	0.31
Vegetable oils (%TEI)	3.9 (2.5)	3.7 (2.3)	3.6 (2.0)	3.7 (2.0)	0.14	3.4 (1.9)	3.4 (2.1)	3.3 (2.1)	2.8 (2.0)	0.01
Olive oil (g/day)	6.4 (4.4)	5.8 (4.8)	5.5 (4.4)	5.8 (4.6)	0.04	7.4 (5.8)	7.2 (5.2)	7.0 (5.4)	5.6 (4.1)	0.003
Peanut oil (g/day)	1.8 (1.6)	1.8 (1.9)	1.6 (1.6)	1.7 (1.4)	0.45	2.4 (1.8)	2.2 (2.1)	2.1 (2.0)	2.1 (1.6)	0.29
Sunflower oil (g/day)	4.9 (3.6)	5 (4.3)	4.9 (3.9)	4.9 (3.5)	0.93	6.1 (4.3)	6.1 (4.6)	6.1 (5.5)	6.1 (4.4)	0.90
Energy intake (MJ/day)	7.7 (2.2)	7.7 (2.6)	7.6 (2.4)	7.2 (2.5)	0.23	10.5 (3.1)	10.4 (3.2)	10.4 (3.2)	10.8 (2.4)	0.42
Vitamin E (mg/day)	11.7 (5.1)	11.6 (5.6)	11.4 (5.8)	11.4 (4.4)	0.73	13.5 (5.0)	13.7 (5.9)	13.7 (6.8)	14.1 (5.4)	0.49
Vitamin C (mg/day)	83.0 (47.5)	89.1 (54.2)	88.8 (55.5)	96.4 (51.5)	0.17	95.5 (56.2)	91.2 (57.1)	92.3 (62.2)	88.5 (56.3)	0.50

Values are medians (IQR). Differences in dietary factors between photoaging grades were assessed using the Kruskal-Wallis test. TEI, Total energy intake.

### Food Composition Table

Food composition was determined using the SU.VI.MAX food composition table [22] with respect to energy and MUFA. This table was compiled from existing tables, notably the French food composition table [23] and recent updates [24], the USDA National Nutrient Database [25] and the British McCance & Widdowson’s food composition table [26], as well as from original publications. In addition, vitamin E and vitamin C intake was estimated using the SU.VI.MAX food composition table. The MUFA intake from each food category for each individual was then estimated as follows. Each complex dish was first broken down into each of its constitutive simple food items (for example, pies into butter, milk…) using a recipe table validated by dietitians. Then, simple food items were grouped into food group, such as vegetable oils, dairy products, meats and processed meats.

### Outcome Variable

The severity of facial skin photoaging was assessed at baseline by trained investigators using a six-grade ordinal scale, each grade being depicted by three photographs to illustrate the diversity and the range of manifestations within each grade [4], [16]. Due to the restricted age range of our population (middle-aged individuals) grades 1 and 6 were rarely present, thus these extreme grades were grouped with grades 2 and 5, respectively. The outcome variable was thus expressed in four grades of severity (grades 1–2, 3, 4 and 5–6).

**Table 3:** Risk of photoaging according to lipid intakes.

Fat intake	Quartile 1	Quartile 2	Quartile 3	Quartile 4	P-value*
**Women (n = 1204)**					
Monounsaturated fat (% TEI)	<13.9	[13.9–15.3[	[15.3–16.7[	≥16.7	
AOR [95% CI]†	1.00 (ref)	0.93 (0.70–1.23)	0.86 (0.65–1.15)	0.88 (0.65–1.19)	0.37
Monounsaturated fat from					
Dairy (%TEI)	<3.5	[3.5–4.3[	[4.3–5.2[	≥5.2	0.12
AOR [95% CI]‡	1.00 (ref)	1.10 (0.84–1.44)	1.13 (0.84–1.51)	1.27 (0.95–1.70)	
Meat (%TEI)	<0.7	[0.7–1.0[	[1.0–1.5[	≥1.5	0.17
AOR [95% CI]‡	1.00 (ref)	1.00 (0.77–1.31)	0.88 (0.67–1.16)	0.84 (0.63–1.12)	
Processed meats (%TEI)	<0.6	[0.6–1.1[	[1.1–1.8[	≥1.8	0.62
AOR [95% CI]‡	1.00 (ref)	0.98 (0.74–1.29)	0.85 (0.65–1.13)	0.95 (0.71–1.26)	
Vegetable oils (% TEI)	<2.7	[2.7–3.7[	[3.7–4.9[	≥4.9	0.009
AOR [95% CI]‡	1.00 (ref)	0.99 (0.75–1.32)	0.92 (0.67–1.25)	0.63 (0.44–0.90)	
Olive oil (g/day)	<3.8	[3.8–5.8[	[5.8–8.4[	≥8.4	
AOR [95% CI]†	1.00 (ref)	0.87 (0.66–1.15)	0.89 (0.65–1.20)	0.69 (0.50–0.95)	0.03
Peanut oil (g/day)	<1.0	[1.0–1.7[	[1.7–2.7[	≥2.7	
AOR [95% CI]†	1.00 (ref)	1.02 (0.77–1.34)	0.91 (0.67–1.24)	0.94 (0.69–1.27)	0.59
Sunflower oil (g/day)	<3.2	[3.2–4.9[	[4.9–7.3[	≥7.3	
AOR [95% CI]†	1.00 (ref)	1.19 (0.90–1.57)	1.12 (0.82–1.52)	1.13 (0.79–1.61)	0.67
**Men (n = 1566)**					
Monounsaturated fat (% TEI)	<13.2	[13.2–14.6[	[14.6–16.0[	≥16.0	
AOR [95% CI]†	1.00 (ref)	0.89 (0.69–1.16)	0.76 (0.58–0.99)	0.76 (0.57–1.00)	0.03
Monounsaturated fat from					
Dairy (%TEI)	<3.2	[3.2–4.0[	[4.0–4.8[	≥4.8	
AOR [95% CI]‡	1.00 (ref)	0.87 (0.67–1.13)	1.10 (0.85–1.42)	1.09 (0.84–1.42)	0.28
Meat (%TEI)	<0.7	[0.7–1.1[	[1.1–1.6[	≥1.6	
AOR [95% CI]‡	1.00 (ref)	1.16 (0.90–1.48)	0.99 (0.77–1.28)	1.00 (0.77–1.30)	0.76
Processed meats (%TEI)	<0.8	[0.8–1.4[	[1.4–2.3[	≥2.3	
AOR [95% CI]‡	1.00 (ref)	1.04 (0.81–1.33)	0.98 (0.75–1.27)	1.09 (0.83–1.44)	0.62
Vegetable oils (% TEI)	<2.4	[2.4–3.3[	[3.3–4.4[	≥4.4	
AOR [95% CI]‡	1.00 (ref)	0.71 (0.54–0.92)	0.61 (0.46–0.81)	0.55 (0.40–0.76)	0.0004
Olive oil (g/day)	<4.7	[4.7–7.1[	[7.1–10.0[	≥10.0	
AOR [95% CI]†	1.00 (ref)	0.84 (0.64–1.09)	0.81 (0.62–1.06)	0.58 (0.43–0.77)	0.0002
Peanut oil (g/day)	<1.4	[1.4–2.2[	[2.2–3.3[	≥3.3	
AOR [95% CI]†	1.00 (ref)	1.10 (0.85–1.42)	0.85 (0.66–1.11)	0.80 (0.61–1.06)	0.09
Sunflower oil (g/day)	<4.0	[4.0–6.1[	[6.1–8.7[	≥8.7	
AOR [95% CI]†	1.00 (ref)	0.84 (0.65–1.09)	0.92 (0.70–1.21)	0.95 (0.68–1.33)	0.99

TEI, Total energy intake,

* Probability of Wald test for linear trend.

† AOR [95% CI]: Adjusted odds ratio and 95% confidence interval adjusted for age, educational level, smoking status, overall physical activity, body mass index, hormonal status, lifetime sun exposure, phototype, geographic location, vitamin E and C intakes and energy.

‡ Adjusted for the same covariates plus total monounsaturated fat intake (%TEI).

### Covariates

Data on age, geographical location (postcode), smoking habits (never, former, current), physical activity (irregular, less than 1h of walking per day, more than one hour of walking per day), educational level (primary school, secondary school or higher education), and hormonal status (non-menopausal, menopausal with use of menopausal hormone therapy, menopausal without use of menopausal hormone therapy) were collected through a self-administrated questionnaire at inclusion. Height and weight were measured using standardised procedures in subjects wearing undergarments. Body mass index (BMI) was calculated as weight (in kilograms) divided by height (in meters, squared), and categorized into three groups: <25 kg/m^2^, 25–30 kg/m^2^, ≥30 kg/m^2^. For geographical location, France was arbitrarily divided into North and South areas using the northern frontier of Aquitaine, Limousin, Auvergne, and Rhône-Alpes regions. In addition, skin phototype was determined at baseline according to the classification proposed by Césarini: I, II, IIIa, IIIb, IV, V, VI [27]. Levels I and VI, which were rare in our population, have been grouped with levels II and V respectively. In addition, lifetime sun exposure was collected using the following question: “How would you describe the intensity of your skin’s exposure to the sun during your lifetime?” none/mild, moderate, or severe [28], [29].

For the present analysis, we included 1264 women and 1655 men aged between 45 and 60 years old, from both placebo and intervention groups, with data for dietary intake and skin photodamage (Fig. 1).

### Statistical Analyses

Separate analyses were conducted for each sex. First, nutrient density was calculated by expressing MUFA as a percentage of total energy intake (TEI) and this nutrient density was then categorized into quartiles. Individual MUFA densities from the main food sources and the intake of the three most frequently consumed oils containing large amounts of MUFA were similarly categorized into quartiles.

Due to the ordinal nature of the outcome variable [30], a partial proportional odds model (PPOM) was used to study the relationship between photoaging and MUFA density independent of total energy intake [31]. The model was adjusted for total energy intake, vitamin E and vitamin C intake, age, and other possible confounders (covariates). Results are expressed as estimated odds ratios (ORs) with their 95% confidence intervals (95% CI) for each quartile with respect to the first quartile as the reference. In addition, a trend for linearity was tested by assigning each subject the median value of their quartile, this value being modeled as a continuous variable. In addition, to study the contributions of specific sources of MUFA independent of total MUFA intake, similar models were performed with respect to the dietary origin of MUFA adjusted on the same covariates, as well as for total MUFA density. Finally, associations between the severity of photoaging and the three most consumed oils (olive oil, sunflower oil and peanut oil) were investigated using the same methods.

All tests were two-sided and type I error was set at P<0.05. Statistical analyses were carried out using SAS® software release 9.1.3 (SAS Institute, Cary, NC, USA).

## Results

The distribution of the women and men enrolled according to photoaging severity and by demographic, medical and behavioral variables is presented in Table 1. As expected, the severity of skin photoaging was strongly linked to age in both sexes, with non-menopausal women presented less severe photoaging. In men, a higher severity of skin photoaging was associated with a lower education level, a higher phototype and a higher latitude (North of France). In both sexes, daily intakes of MUFA were lower among the most severe grades of photoaging than among the lowest grades of photoaging (Table 2). Similar associations were found for intake of MUFA from vegetable oils and olive oil.

After adjustment for possible cofounders, a significant association was found in men between severity of photoaging and dietary intake of MUFA (Table 3). Higher intake of MUFA was associated with a lower risk of severe photoaging (highest *vs* lowest quartile of MUFA: AOR = 0.76, 95%CI (0.57–1.00), p_ = _0.03). For both sexes, a higher consumption of MUFA provided by vegetable oils was found to be associated with a lower risk of severe photoaging (for women: 0.63 (0.44–0.90), p = 0.01; for men: 0.55 (0.40–0.76), p = 0.0004). No association was found with MUFA intake from dairy products, meats and processed meats. Finally, of the three most frequently consumed oils (sunflower, olive and peanut oil), a significant association was found for olive oil. A higher intake of olive oil was significantly associated with a lower risk of severe photoaging (for women: 0.69 (0.50–0.95), p = 0.03; for men: 0.58 (0.43–0.77), p = 0.0002). In our population, olive oil was the main source of vegetable oil MUFA (59% and 51%, respectively), whereas sunflower and peanut oils provided only 15% and 13% of vegetable oil MUFA.

## Discussion

In this study we report a significant association between total intake of MUFA and skin photoaging in men but not in women. When the individual contribution of each source of MUFA was considered, higher intakes of MUFA from vegetable oil were however found to be negatively associated with severe skin photoaging independently of environmental factors known to cause premature and accelerated skin aging in both sexes, whereas intake of MUFA from animal products (dairy products, meat and processed meats) was not significantly associated with skin photoaging. In particular, a higher consumption of olive oil was inversely correlated with the severity of skin photoaging. Olive oil was the only one of the three vegetable oil sources of MUFA usually consumed in our study population (olive oil, sunflower oil and peanut oil) to present such a protective effect. These findings are consistent with previous studies which have addressed individual aspects of this relationship [32]–[34]. Hence, Purba et al. [33] reported a negative association between total MUFA intake, olive oil intake and skin aging, whereas Nagata et al. [34] found a positive association between MUFA intake and skin elasticity. In contrast, another study did not find any association between oleic acid consumption and wrinkled appearance and even reported a higher risk of senile dryness in higher consumers [32]. However, in these two last studies, fatty acids were considered as a whole, without taking into account their specific origin.

The observed negative association between olive oil intake and severe photoaging may be due to its specific fatty acid profile with a high amount of MUFA and a low ratio of n-6 PUFA/n-3 PUFA [35], [36]. Indeed, MUFA is far less susceptible to peroxidation than PUFA. In contrast to olive oil, we did not find dairy products to be negatively associated with skin photodamage although they provide comparably high amounts of MUFA to olive oil. However, dairy products are also a rich source of saturated fatty acids, which are known to be associated with insulin resistance and an increase of inflammatory processes [37]. Another hypothesis would be that squalene and polyphenols contained in olive oils may play a role in preventing photodamage [35], [36]. Squalene is to a large extent sequestered in the skin (sebum is reported to contain 12%), where it is believed to exert a major protective effect against free radical damage and skin dryness [36]. Polyphenols are also known to be powerful radical scavengers. Both squalene and polyphenols have been assumed to be primarily responsible for the beneficial effects of the Mediterranean diet. Finally, as expected, the consumption of olive oil in our population was also positively associated with high consumption of fruits, vegetables, fish and tea, and negatively associated with sweet products, butter and milk. In that sense, the consumption of olive oil could also be considered as a marker of a healthy diet [38].

Our study has both strengths and limitations. The strengths encompass the assessment of dietary intake based on a mean of ten computerized 24-hour diet records in order to take into account weekly and seasonal intra-individual variability in the intake of the monounsaturated fatty acids, which may be considerable [21]. Furthermore, a validated manual containing a photographic guide was provided to each subject in order to facilitate estimation of portion size among seven proposed portion sizes [19]. Our study was also a well-characterized sample of middle-aged women and men living in the community evaluated. Finally, due to the number of statistical tests performed, we cannot exclude that some significant associations found may be due to chance, in spite of the overall coherence between findings in men and in women. Moreover, the cross-sectional design of our study does not allow us to address the causality of the associations observed.

In conclusion, our findings provide support for a beneficial role of olive oil in preventing severe facial photoaging. This result should be supported by further mechanistic studies taking into account the relationship between a diet rich in MUFA, subcutaneous fat and the overall aging process, of which skin photodamage may be a readily observable surrogate marker. Finally, our findings provide a useful insight into the beneficial effect of olive oil, as the main source of dietary fat, as promoted in a diversified diet, although the only way to demonstrate that olive oil can prevent photodamage is to perform an interventional study.
